# Assessing Metabolic Syndrome Risk in Children and Adolescents with Prader–Willi Syndrome: A Comparison of Index Performance

**DOI:** 10.3390/jcm14134716

**Published:** 2025-07-03

**Authors:** Graziano Grugni, Fiorenzo Lupi, Mirko Bonetti, Sarah Bocchini, Carmen Bucolo, Domenico Corica, Antonino Crinò, Maria Felicia Faienza, Danilo Fintini, Maria Rosaria Licenziati, Mohamad Maghnie, Enza Mozzillo, Roberta Pajno, Giuseppe Zampino, Alessandro Sartorio, Giorgio Radetti

**Affiliations:** 1 Experimental Laboratory for Auxo-Endocrinological Research, Istituto Auxologico Italiano, Istituto di Ricovero e Cura a Carattere Scientifico (IRCCS), Piancavallo, 28824 Verbania, Italy; sartorio@auxologico.it; 2 Division of Neonatology/NICU, Hospital of Bolzano (SABES-ASDAA), Teaching Hospital of Paracelsus Medical University (PMU), 39100 Bolzano, Italy; fiorenzo.lupi@sabes.it; 3 Observatory for Health–Health Department Province of Bolzano, 39100 Bolzano, Italy; mirko.bonetti@provincia.bz.it; 4 Prader–Willi Reference Center, Endocrinology and Diabetology Unit, Bambino Gesù Children Hospital, IRCCS, 00165 Rome, Italy; sarah.bocchini@opbg.net (S.B.); danilo.fintini@opbg.net (D.F.); 5 Pediatric Unit, San Raffaele Institute, IRCCS, 20132 Milan, Italy; bucolo.carmen@hsr.it (C.B.); pajno.roberta@hsr.it (R.P.); 6 Pediatric Unit, Department of Human Pathology of Adulthood and Childhood “G. Barresi”, University of Messina, 98124 Messina, Italy; domenico.corica@unime.it; 7 Rare Disease Unit, Fondazione Policlinico Universitario A. Gemelli, IRCCS, 00136 Rome, Italy; antonino.crino@guest.policlinicogemelli.it (A.C.); giuseppe.zampino@policlinicogemelli.it (G.Z.); 8 Pediatric Unit, Department of Precision and Regenerative Medicine and Ionian Area, University of Bari “Aldo Moro”, 70124 Bari, Italy; mariafelicia.faienza@uniba.it; 9 Neuro-Endocrine Diseases and Obesity Unit, Department of Neurosciences, Santobono-Pausilipon Children’s Hospital, 80139 Naples, Italy; m.licenziati@santobonopausilipon.it; 10 Department of Pediatrics, Istituto Giannina Gaslini, IRCCS, 16147 Genova, Italy; mohamadmaghnie@gaslini.org; 11 Department of Neuroscience, Rehabilitation, Ophthalmology, Genetics, Maternal and Child Health, University of Genova, 16147 Genova, Italy; 12 Section of Pediatrics, Department of Translational Medical Science, Federico II University of Naples, 80131 Naples, Italy; enza.mozzillo@unina.it; 13 Marienklinik, 39100 Bolzano, Italy; giorgio.radetti@marienklinik.it

**Keywords:** Prader–Willi syndrome, metabolic syndrome, Triglycerides/HDL-Cholesterol ratio

## Abstract

**Background:** Currently, there is a lack of data regarding the reliability of different anthropometric, instrumental, and biochemical indexes in detecting metabolic syndrome (MetS) in pediatric patients with Prader–Willi syndrome (PWS). Therefore, this study aimed to compare the accuracy of different indices to identify the simplest and most accurate predictor of MetS in this at-risk population. **Methods:** We conducted a multicenter study involving 124 children and adolescents with PWS (61 males and 63 females), aged 13.6 ± 3.7 years. For each participant, we assessed all components of MetS, defined according to either the Identification and prevention of Dietary- and lifestyle-induced health EFfects In Children and infantS (IDEFICS) study or the International Diabetes Federation (IDF) criteria, based on age. The following indexes were calculated: Body Mass Index (BMI), BMI standard deviation score (BMI-SDS), tri-ponderal mass index, body mass fat index, fat mass index, fat-free mass index, body shape index, visceral adiposity index, waist-to-height ratio, cardiometabolic index, total cholesterol/high-density lipoprotein cholesterol (HDL-C) ratio, and triglycerides/HDL-C (TG/HDL-C) ratio. **Results:** MetS was identified in 24 subjects (9 females and 15 males), representing 19.4% of the sample. When comparing the receiver operating characteristic (ROC) curves, the TG/HDL-C ratio and cardiometabolic index demonstrated significantly better performance than the other indices in detecting MetS, with no difference between the two. As a result, we focused on the TG/HDL-C ratio since it is the simplest measure, requiring no additional anthropometric data compared to the cardiometabolic index. Additionally, applying age- and gender-specific thresholds can further improve its accuracy. **Conclusions:** The TG/HDL-C ratio, which requires only two standard biochemical markers, provides the same accuracy as more complex indexes in detecting MetS in children and adolescents with PWS, making it the optimal predictor for MetS in this population.

## 1. Introduction

Prader–Willi syndrome (PWS) is a rare disorder caused by the loss of expression of a cluster of paternally active genes in the PWS region on chromosome 15 (15q11–13). About 65–70% of PWS patients result from a paternal deletion (del15), 25–30% from maternal uniparental disomy for chromosome 15 (UPD15), and the remaining cases are caused by imprinting defects, chromosome 15 translocations, or inversions [1]. PWS represents the first human disorder linked to genomic imprinting and is considered the most common syndromic cause of life-threatening obesity. The estimated prevalence of PWS is approximately 1 in 21,000 newborns [2].

PWS patients typically present with severe neonatal hypotonia, poor sucking reflex, and failure to thrive, followed by hyperphagia, leading to early-onset childhood obesity and associated comorbidities if left uncontrolled. Clinical features include dysmorphic abnormalities, behavioral disorders, cognitive impairment, psychosis, short stature, and multiple endocrine deficiencies [3,4,5]. A complex hypothalamic–pituitary dysfunction is believed to be responsible for many signs and symptoms of this multifaceted phenotype [6].

Life expectancy for patients with PWS is reduced compared to the general population [7], with increased morbidity and mortality primarily linked to the clinical impact of obesity [8,9]. In non-PWS subjects, obesity is frequently associated with metabolic syndrome (MetS), which represents a relevant risk factor for the development of type 2 diabetes mellitus (T2DM) and cardiovascular diseases (CVDs) [10]. Given that T2DM and CVD are leading causes of morbidity and mortality worldwide, it is conceivable that MetS contributes to the pathogenesis of early morbidity and mortality in PWS. While the metabolic profile of PWS patients is usually more favorable than that of individuals with simple obesity, due to lower visceral adiposity [11,12], previous research has shown that obese individuals with PWS had a similar prevalence of MetS to that of obese controls, both in pediatric and adult populations [13,14]. Interestingly, MetS was also found in non-obese subjects with PWS, albeit at a lower frequency [14]. Therefore, early recognition and treatment of MetS could improve both health and life expectancy in these patients.

In recent years, various indexes have been proposed to detect metabolic risk and identify MetS. Our previous work demonstrated that Body Mass Index (BMI), the most easily calculated index, performed similarly to other adiposity indexes such as fat mass index (FMI) [15], tri-ponderal mass index (TMI) [16], and body mass fat index (BMFI) [17], as well as indexes assessing body fat distribution like waist-to-height ratio (WtHR) [18] and nutritional status such as fat-free mass index (FFMI) [19], in a sample of 1332 obese children and adolescents [17]. Furthermore, we found that the visceral adiposity index (VAI) [20], cardiometabolic index (CMI) [21] and triglycerides/high-density lipoprotein cholesterol (TG/HDL-C) ratio [22] performed better than BMI, BMI standard deviation score (SDS), TMI, and WtHR in identifying MetS in a large cohort of children and adolescents with severe obesity (BMI SDS > 3.00) [23]. In individuals with PWS, we recently showed that BMI was the most reliable predictor of MetS in adult subjects when compared to other adiposity and body composition indexes [24]. However, there is currently no data on the reliability of different anthropometric, instrumental, and biochemical indexes for identifying MetS in children and adolescents with PWS.

With this background, the objective of the present study was to compare the accuracy of various indexes in identifying MetS in a cohort of patients aged 7 to 18 years with genetically confirmed PWS.

## 2. Materials and Methods

### 2.1. Study Population

A retrospective cohort study was performed, enrolling 124 subjects with PWS [61 females and 63 males, aged 13.6 ± 3.7 years (mean ± SD), with a BMI-SDS of 2.13 ± 1.42]. Participants were enrolled between June 2021 and January 2024 from 9 Italian PWS reference centers. All patients showed the typical PWS clinical phenotype [3]. Sixty-five subjects had del15, while UPD15 was found in forty-seven patients. One female had a chromosome 15 translocation, and a positive methylation test was demonstrated in the remaining eleven patients with PWS, but the underlying genetic defect was not identified.

At the time of the study, four subjects were receiving treatment for arterial hypertension, nine patients were on antidiabetic medications, and one female was being treated for hyperlipidemia. Six subjects had central hypothyroidism and were biochemically euthyroid on thyroxine replacement. Nine females and nine males were undergoing sex steroid replacement therapy, while fifty-six patients were receiving recombinant human growth hormone (rhGH) therapy, and fifty-five had previously received rhGH. Thirteen patients had never received rhGH therapy. Following international recommendations [25] and the standardized Diagnostic and Therapeutic Care Pathway for PWS in Italy [26], both lean and obese patients followed a low-calorie diet.

Pubertal development was assessed according to Tanner’s stages [27]; 13 patients were prepubertal (stage 1), 93 were pubertal (stages 2–4), and 1 female had reached full sexual maturity (stage 5).

The study protocol was approved by the Ethical Committee (EC) of Istituto Auxologico Italiano, Milan, Italy (EC code: 2021_02_23_10; date of approval: 23 February 2021; research project code: 01C125; acronym: PWSPEDIPM). Written informed consent was obtained from parents, and written assent was provided by the children and adolescents when appropriate. All procedures complied with the Helsinki Declaration of 1975, as revised in 2008, and the European Convention on Human Rights and Biomedicine for Research involving Children. They adhered to Directive 95/46/EC of the European Parliament and of the Council of 24 October 1995 regarding data protection and confidentiality.

### 2.2. Anthropometric Data

All subjects underwent body measurements in a fasting state after voiding. The physical examination included measurements of height, weight, and waist circumference (WC) by trained investigators, according to the Anthropometric Standardization Reference Manual [28]. Standing height was measured using a Harpenden Stadiometer (Holtain Limited, Crymych, Dyfed, UK). Body weight was measured to the nearest 0.1 kg using accurate and calibrated standard beam scales. WC was measured at the midpoint between the lowest rib and the top of the iliac crest after a gentle expiration, using a non-elastic flexible tape measure.

### 2.3. Blood Pressure Measurements and Instrumental Examination

Systolic and diastolic blood pressure (BP) were measured to the nearest 2 mmHg in the supine position after 5 min of rest using a mercury sphygmomanometer with an appropriately sized cuff [29]. The average of three measurements was used for analysis.

Fat mass (FM), fat mass percentage (FM%), fat-free mass (FFM), and fat-free mass percentage (FFM%) were assessed using dual-energy X-ray absorptiometry (DXA), as previously described [30].

### 2.4. Laboratory Analyses

Blood samples were drawn after a 12 h overnight fast to assess blood glucose, insulin, hemoglobin A1c (HbA1c), total cholesterol (TC), HDL-C, and triglycerides (TGs), which were measured using standard enzymatic methods. Insulin resistance (IR) was calculated using the homeostatic model assessment for insulin resistance (HOMA-IR) [31].

### 2.5. Definitions

Standardized age- and gender-specific weight, height, and BMI percentiles from the World Health Organization (WHO) were used to calculate SDS and classify subjects with PWS as non-obese (BMI < 2.0 SDS) or obese (BMI > 2.0 SDS) [32].

Different criteria have been used to define MetS based on age groups (Table 1). For children aged from 7 to 10 years, MetS was defined according to the Identification and prevention of Dietary- and lifestyle-induced health EFfects In Children and infantS (IDEFICS) study [33], with at least three of the following criteria: WC > 90th percentile [34]; systolic BP or diastolic BP > 90th percentile by gender and age [35]; TG > 90th percentile or HDL-C < 10th percentile by gender and age [36]; HOMA-IR > 90th percentile or fasting blood glucose > 90th percentile by gender and age [37]. According to the International Diabetes Federation (IDF) criteria for MetS diagnosis in subjects older than 10 years [38], our patients were considered to have the MetS if they had abdominal obesity [WC > 90th percentile for ages < 16 years [39], and >94 cm for males and >80 cm for females for ages > 16 years] plus two or more of the following factors: (1) raised TG level, >150 mg/dL (1.7 mmol/L), for ages < 16 years and the same cutoff or specific treatment for this lipid abnormality for ages > 16 years; (2) reduced HDL-C, <40 mg/dL (1.03 mmol/L) for males and females for ages < 16 years and <40 mg/dL for males and <50 mg/dL (1.29 mmol/L) for females, or specific treatment for this lipid abnormality for ages > 16 years; (3) raised BP, systolic BP > 130 mmHg or diastolic BP > 85 mmHg for ages < 16 years and the same cutoff or treatment of previously diagnosed hypertension for ages > 16 years; (4) raised fasting plasma glucose (FPG) concentration > 100 mg/dL (5.6 mmol/L) or previously diagnosed impaired glucose tolerance/T2DM for all ages.

The following indexes were calculated according to the respective formulas:

1. BMI: weight (kg)/height in m^2^.
2. BMI SDS [32]: [BMI-mean BMI (for age and gender)]/SD.
3. TMI [16]: mass in kg/height in m^3^.
4. BMFI [17]: BMI × FM (%) × WC (cm).
5. FMI [15]: fat mass in kg/height in m^2^.
6. FFMI [19]: fat-free mass in kg/height in m^2^.
7. Body Shape Index (ABSI) [40]: WC/BMI2/3 × height1/2.
8. VAI [20]: males: [WC/39.68 + (1.88 × BMI)] × (TG/1.03) × (1.31/HDL); females: [WC/36.58 + (1.89 × BMI)] × (TG/0.81) × (1.52/HDL).
9. WtHR [18]: WC (cm)/height (cm).
10. CMI [21]: WtHR × TG/HDL-C.
11. TC/HDL-C ratio [41].
12. TG/HDL-C ratio [22].

### 2.6. Statistical Analysis

Based on the box-plot method, no outliers have been identified for any of the observed variables; furthermore, no patients were excluded from the analysis. The Kolmogorov–Smirnov goodness-of-fit test was used to assess the normality of the distribution of each continuous variable. All tested variables were non-normally distributed, as confirmed by the analysis of the distribution graphs. Continuous data were presented as medians (interquartile ranges, IQRs), whereas median values were tested for statistical significance using a two-tailed Wilcoxon test. An adjusted receiver operating characteristic (ROC) analysis using clinical cut points for metabolic risks was performed across the different variables. ROC curves were generated to obtain the values of the area under the curve (AUC), along with sensitivity, specificity, and 95% CI [42]. Values of AUC between 0.7 and 0.9 must be considered moderately predictive, while values between 0.9 and 1 are highly predictive. The Youden index was calculated to identify the optimal cutoff, as it is often applied as a summary of the ROC curve and is a practical, validated measure to assess the performance of a diagnostic test [43,44]. Thus, predictive validity and optimal cutoff values were analyzed by ROC curves, the area under the curves (AUC), and the largest Youden’s index. In addition, positive likelihood ratio (PLR), negative likelihood ratio (NLR), positive predictive value (PPV), and negative predictive value (NPV) were examined. Spearman correlation coefficients were calculated to assess the relationship between the TG/HDL-C ratio and metabolic characteristics.

The significance threshold was set at *p* < 0.05. The data were analyzed using SAS Enterprise Guide 7.1 (SAS Institute Inc., Cary, NC, USA).

## 3. Results

According to BMI cutoffs, 70 subjects with PWS were classified as obese (29 females) and 54 as non-obese (32 females). All obese patients had central obesity, while WC was pathological in 36 non-obese subjects (26 females). Twenty-nine patients (eleven females) had arterial hypertension. Eighteen individuals (six females) had hypertriglyceridemia, while twenty-two subjects (thirteen females) had a low HDL-C level. Twenty-three subjects (eight females) had altered glucose metabolism. The presence of MetS was found in 24 subjects (9 females) (19.4%). Non-obese subjects with PWS exhibited a lower frequency of MetS (5/54: 9.2%) as compared to the obese group (19/70: 27.1%) (*p* < 0.05).

The clinical and laboratory characteristics of the entire study group, subdivided by gender, are reported in Table 2. As expected, boys were taller than girls, while there were no significant differences in weight and BMI. Girls exhibited a worse body composition than boys, characterized by a higher fat mass percentage and a lower percentage of fat-free mass. In contrast, boys had higher glycemia, TMI, FFMI, ABSI, and TC/HDL-C ratio than girls.

Table 3 reports the clinical and laboratory parameters of the study group according to the presence of MetS (MetS +) or its absence (MetS −). As expected, the clinical data and metabolic profile were significantly worse in the MetS+ group, except for HbA1c, total cholesterol, and systolic blood pressure, which did not differ between the two subgroups. Subjects with MetS + had higher BMFI, FMI, VAI, WtHR, CMI, TC/HDL-C, and TG/HDL-C ratios than individuals with MetS −.

The comparison between the two genders, based on the presence or absence of MetS, is presented in Table 4. No difference was found between males and females affected by MetS (MetS +). By contrast, subjects without MetS (MetS −) showed a worse body composition in girls, characterized by a higher percentage of fat mass and a lower percentage of fat-free mass. Furthermore, boys had a higher FFMI and lower VAI than girls, while the latter showed a lower TC/HDL-C ratio.

**Figure 1:**
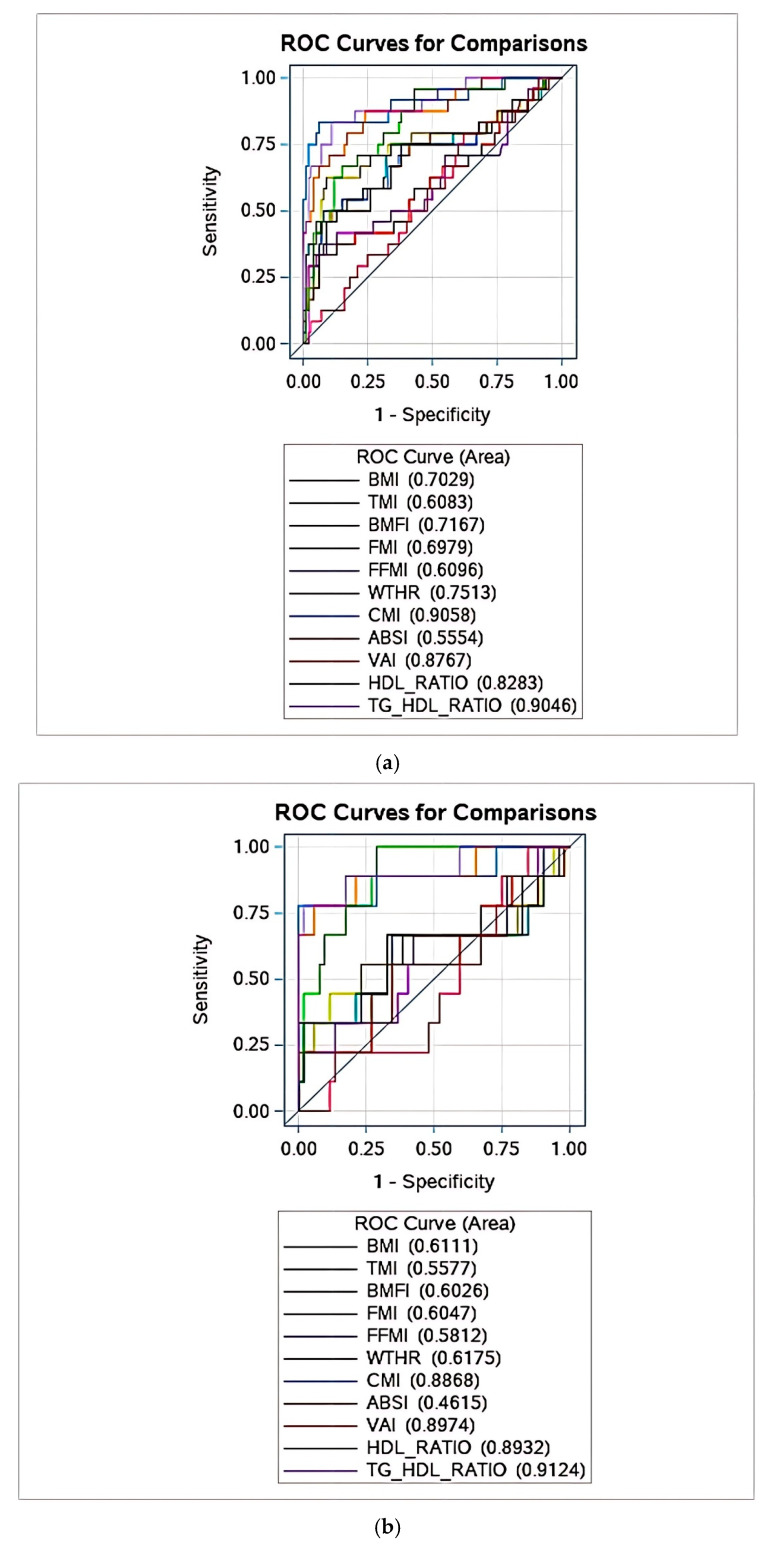

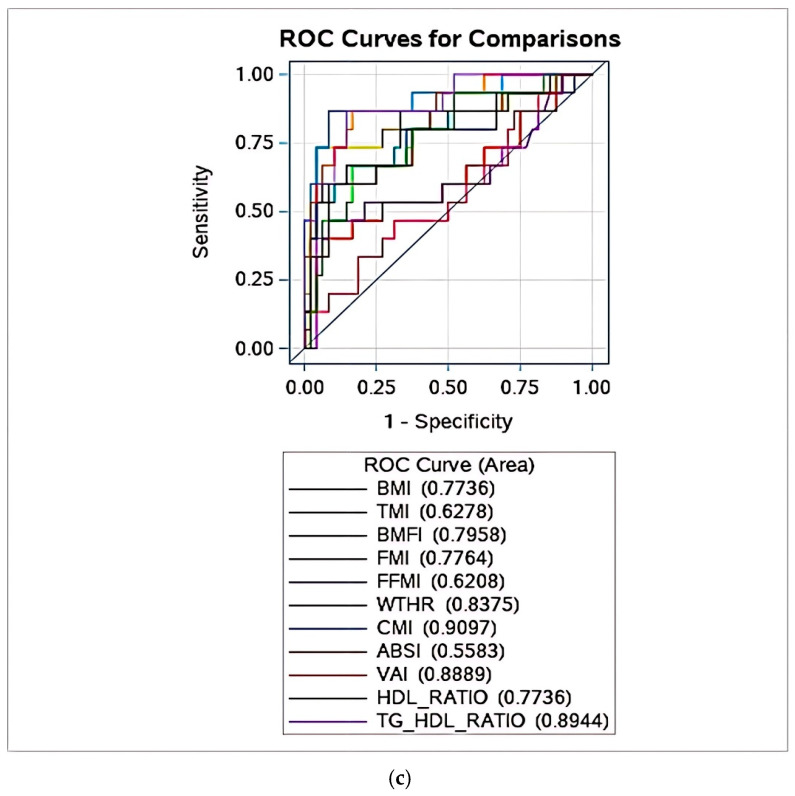
(**a**) ROC curves for the whole study group are reported, showing the best performance of CMI and TG/HDL-C ratio in identifying MetS. No difference was found between these two indexes. Abbreviations: BMI: Body Mass Index; TMI: tri-ponderal mass index; BMFI: body mass fat index; FMI: fat mass index; FFMI: fat-free mass index; WtHR: waist-to-height ratio; CMI: cardiometabolic index; ABSI: body shape index; VAI: visceral adiposity index; TC/HDL-C: total cholesterol/HDL-C ratio; TG/HDL-C: triglycerides/HDL-C ratio. (**b**) ROC curves in females are reported, showing the best performance of CMI and TG/HDL-C ratio in identifying MetS. No difference was found between these two indexes. Abbreviations: BMI: Body Mass Index; TMI: tri-ponderal mass index; BMFI: body mass fat index; FMI: fat mass index; FFMI: fat-free mass index; WtHR: waist-to-height ratio; CMI: cardiometabolic index; ABSI: body shape index; VAI: visceral adiposity index; TC/HDL-C: total cholesterol/HDL-C ratio; TG/HDL-C: triglycerides/HDL-C ratio. (**c**) ROC curves in males are reported, showing the best performance of CMI and TG/HDL-C ratio in identifying MetS. CMI showed a greater diagnostic value than the TG/HDL-C ratio. Abbreviations: BMI: Body Mass Index; TMI: tri-ponderal mass index; BMFI: body mass fat index; FMI: fat mass index; FFMI: fat-free mass index; WtHR: waist-to-height ratio; CMI: cardiometabolic index; ABSI: body shape index; VAI: visceral adiposity index; TC/HDL-C: total cholesterol/HDL-C ratio; TG/HDL-C: triglycerides/HDL-C ratio.

Figure 1a–c report the ROC curves for the whole study group, boys and girls, respectively, showing the best performance of the TG/HDL-C ratio and CMI in identifying MetS, with no difference between these two indexes.

Table 5 reports the sensitivity, specificity, PPV, and NPV for identifying MetS, together with the Youden Index and the Area Under the Receiver Operating Characteristic curve (AUROC), for CMI and the TG/HDL-C ratio. The analysis stratified by gender revealed no differences between the two indexes for females, whereas CMI demonstrated greater diagnostic accuracy in males, as measured by the AUROC. Notably, the optimal cutoff value, as determined by the Youden index, remained unchanged. In contrast, for the TG/HDL-C ratio, males had a reduced diagnostic accuracy (AUROC), while the cutoff value (Youden) remained consistent. Considering the entire study group, no significant difference was found between the two indexes for any of the parameters. Consequently, the TG/HDL-C ratio was chosen as the preferred index due to its simplicity, because it did not require any additional anthropometric measures compared to CMI.

Table 6 shows the correlation between the TG/HDL-C ratio and the components of MetS, both in the whole study group and separately by gender. Considering all 124 patients, a significant correlation was found for all clinical and biochemical parameters of MetS. The same results were observed in both genders, except for serum glucose level and systolic and diastolic BP (males only). As expected, a highly significant correlation was found between the TG/HDL-C ratio and both HDL-C and triglycerides, since this ratio includes the same variables.

**Figure 2:**
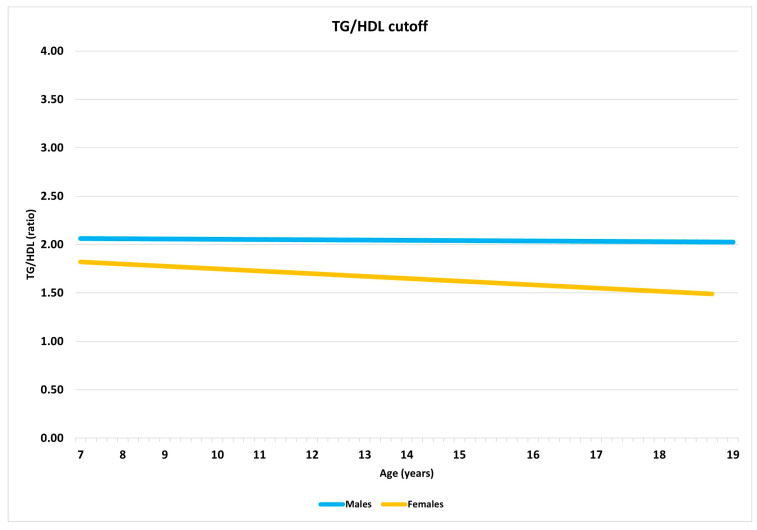
Trend of TG/HDL-C ratio cutoff for MetS, according to age and sex. The mean difference in cutoff values between the sexes calculated at all ages was not significant. Abbreviation: TG/HDL-C: triglycerides/HDL-C ratio.

Figure 2 presents the trend of the TG/HDL-C ratio by age and gender. The mean difference in the cutoff values between the two genders, calculated across all ages, was not significant.

## 4. Discussion

The mortality rate among patients with PWS is estimated to be higher than that of the general population, with estimates of 3% per year [45] and reports suggesting it is 15 times higher than in the general population [7]. This elevated mortality is mainly attributed to the higher incidence of heart problems, T2DM, arterial hypertension, and respiratory complications in PWS [7,8]. However, recent advances in early diagnosis and multidisciplinary care, including rGH therapy, have substantially increased life expectancy for patients with PWS [46]. Consequently, it is likely that, in the future, the prevalence of age-related diseases, including cardiovascular disease and impaired glucose and lipid metabolism, will become more pronounced. Since MetS is strongly associated with both atherosclerotic CVD and T2DM [10], its prevalence in individuals with PWS is expected to rise.

In this context, we previously found the presence of MetS in 37.5% of adults with PWS [24], with a slight increase compared to those observed 7 years earlier (34.2%) [14]. In this study, MetS affected 9.2% of non-obese children and adolescents with PWS and 27.1% of obese individuals. Again, these values are significantly higher than those reported in our previous study, in which we found that MetS was present in 15.6% of obese subjects and none of the non-obese subjects [13].

Overall, these data highlight the importance of the early identification of MetS or the risk of developing MetS in the population with PWS to prevent severe clinical consequences and reduce early mortality in such patients. For this purpose, in the present study, we have compared a group of surrogate indexes based on anthropometric data, body composition, and routine biochemical parameters in a group of 124 children and adolescents with PWS to identify which is the simplest and most reliable for identifying MetS. Our analysis revealed that the TG/HDL-C ratio and CMI performed significantly better than other indicators such as BMI, BMI-SDS, TMI, BMFI, FMI, FFMI, ABSI, VAI, WtHR, and TC/HDL-C, with no substantial difference between them. These results align with our previous findings in a large cohort of pediatric subjects with simple obesity [23], except for the VAI, which showed poor predictive value in subjects with PWS. Considering all 124 patients with PWS, TG/HDL-C ratio and CMI demonstrated similar values in terms of sensitivity, specificity, PPV, NPV, Youden index, and AUROC. However, when assessing the two genders separately, we observed that CMI had a higher diagnostic accuracy for MetS in males compared to the TG/HDL-C ratio, as measured by AUROC. In this context, however, operator-related variability in WC determination and the difficulty of obtaining a reproducible measurement in obese patients must be taken into account. In light of this, we do not consider a gender-specific recommendation to be warranted. Given its simplicity, we ultimately selected the TG/HDL-C ratio as the preferred method.

In our study, we found a significant correlation between the TG/HDL-C ratio and the clinical and biochemical components of MetS. Altogether, these findings suggest that the TG/HDL-C ratio is a reliable tool for detecting MetS in children and adolescents with PWS, consistent with observations in the general population [23,47,48]. It is also noteworthy that our group of patients with PWS included both obese and non-obese subjects, which further strengthens the relevance of these results. Our data seem to align with previous studies in non-PWS subjects, where the TG/HDL-C ratio has proven useful for identifying a worsened cardiometabolic profile in both obese and non-obese children and adolescents [49].

Previous studies evaluating the predictive value of the TG/HDL-C ratio for cardiometabolic risk and MetS have shown different cutoff values in different populations. In obese adolescents, the highest values for TG/HDL-C ratio (>1.80) was closely correlated with the higher risk of MetS [48]. Data from the Healthy study, which assessed the prevalence of severe obesity and associated risk in a cohort of 6,365 students, estimated a high TG/HDL-C ratio as 3.14 [50]. Di Bonito et al. demonstrated that in children with a high prevalence of overweight, a TG/HDL-C ratio > 2.0 had a two- to three-fold increased risk of left ventricular concentric hypertrophy, compared to those with a TG/HDL-C ratio < 2.0. [49]. A nationwide population-based study in Korea showed that a TG/HDL-C ratio > 2.6 had the highest predictability for cardiometabolic risk factor clustering [51]. In type 1 diabetes, patients with non-albuminuric mildly reduced estimated glomerular filtration phenotype showed higher levels of median TG/HDL-C ratio (1.08) when compared to the reference category [52]. These discrepancies are likely related to the different clinical characteristics of the study groups, including the type of disease and its severity, age, sample size, familial components, and ethnic origin.

In our cohort of children and adolescents with PWS, the thresholds for identifying the risk of MetS using the TG/HDL-C ratio based on age and gender tended to decrease over time, particularly in girls. This contrasts with our previous findings in children and adolescents with simple obesity, where the thresholds showed a clear increase over the years [23]. Furthermore, no significant gender differences were observed. These discrepancies might be attributed to the different characteristics of the study populations, given the various weight statuses of the subjects with PWS enrolled in the present study, compared to the high degree of obesity of the patients included in our previous investigation. Moreover, the peculiar lipid profile of children and adolescents with PWS, characterized by higher HDL-C compared to matched controls, should also be considered [53]. However, this tendency of the cutoff to change with age highlights the importance of accurately interpreting the predictive capacity of the TG/HDL-C ratio for MetS.

A strength of this study is that the recruitment and examination of children and adolescents with PWS were performed by experienced and well-trained medical staff from PWS reference centers belonging to the Italian Network for Rare Diseases. In addition, body composition was assessed using DXA, a highly accurate technique.

However, there were some limitations. The multicenter nature of this study made data interpretation more challenging, although standardized procedures such as anthropometric, clinical, and instrumental assessments were used across centers. Although hematological analyses were performed in different laboratories, all centers belong to the Italian National Health System and undergo semi-annual quality controls and inter-laboratory comparisons, thus contributing to limiting the potential differences between laboratories. Furthermore, the retrospective design of the study and the small sample size, especially in gender-stratified subgroups, due to the rarity of PWS, limited the statistical power of the analysis and its external validity. In this respect, the small number of subjects with MetS determined high specificity values and very wide confidence intervals. Additionally, the absence of a control group due to ethical reasons is another limitation. Finally, the cross-sectional design of the study does not allow insights into the natural history of MetS in PWS, or how it evolves over time.

Despite these limitations, our results provide valuable insights into the metabolic profile of children and adolescents with PWS. The TG/HDL-C ratio and CMI were found to be the most reliable tools for detecting MetS in this population, with the TG/HDL-C ratio being the more straightforward and more cost-effective option. In this context, it is worth noting that the TG/HDL-C ratio does not necessitate the assessment of body composition, and therefore, it leads to a reduction in costs. Finally, our findings suggest that the accuracy of the TG/HDL-C ratio can be further improved by using age- and gender-appropriate thresholds to ensure timely diagnosis of MetS. According to the standardized diagnostic and therapeutic pathway for PWS in Italy [26], the suggested interval for metabolic screening in the care of children and adolescents, including the detection of MetS, is one year. In this context, the use of the TG/HDL-C ratio, both in obese and non-obese subjects, is recommended as a practical tool to be considered in current screening guidelines.

In conclusion, our study could contribute to optimizing the clinical management of PWS, with the TG/HDL-C ratio emerging as a simple and reliable method for early detection of MetS. However, further studies with larger sample sizes are needed to confirm these findings. Furthermore, it is essential to emphasize the need for prospective longitudinal studies to validate the predictive performance of the considered indices and to gain a deeper understanding of the evolution of MetS in children and adolescents with PWS.

## Figures and Tables

**Table 1:** Age-related criteria for the diagnosis of MetS adopted in the study.

Age	Criteria	WC	SBP or DBP	Lipids		Glucose Metabolism	Diagnosis of MetS
7– < 10 years	IDEFICS [33]	>90th percentile	>90th percentile	TG > 90th percentile or HDL-C < 10th percentile		HOMA-IR > 90th percentile or fasting blood glucose > 90th percentile	At least three factors
10– < 16 years	IDF [38]	>90th percentile	SBP > 130 mmHg or DBP > 85 mmHg	TG > 150 mg/dL (1.7 mmol/L)	HDL-C < 40 mg/dL (1.03 mmol/L)	FPG > 100 mg/dL (5.6 mmol/L) or previously diagnosed IGT/T2DM	Abdominal obesity plus two or more of the other factors
16+ years	IDF [38]	>94 cm for males and >80 cm for females	SBP > 130 mmHg or DBP > 85 mmHg, or specific treatment	TG > 150 mg/dL (1.7 mmol/L) or specific treatment	HDL-C < 40 mg/dL for males and <50 mg/dL (1.29 mmol/L) for females, or specific treatment	FPG > 100 mg/dL (5.6 mmol/L) or previously diagnosed IGT/T2DM	Abdominal obesity plus two or more of the other factors

Abbreviations: IDEFICS: Identification and prevention of Dietary- and lifestyle-induced health EFfects In Children and infantS study; IDF: International Diabetes Federation criteria; WC: waist circumference; SBP: systolic blood pressure; DBP: diastolic blood pressure; TG: triglyceride; HDL-C: high-density lipoprotein; HOMA-IR: homeostatic model assessment for insulin resistance; FPG: fasting plasma glucose; IGT: impaired glucose tolerance: T2DM: type 2 diabetes mellitus; MetS: metabolic syndrome.

**Table 2:** Clinical and biochemical data by gender. Data are expressed as median (interquartile range).

Variable	Total (n = 124)	Females (n = 61)	Males (n = 63)	*p*
Age (yr)	13.3 (6.9–18.9)	13.2 (7.0–18.9)	14.0 (6.9–18.8)	0.6331 ^$^
Weight (kg)	60.1 (19.0–121.7)	57.8 (19.0–121.7)	68.0 (23.0–120.6)	0.0921 ^$^
Height (cm)	149.3 (108.0–174.7)	144.0 (108.0–173.1)	153.2 (110.1–174.7)	0.0058 ^$^ *
WC (cm)	86.5 (54.0–131.0)	83.0 (54.0–125.0)	93.0 (56.0–131.0)	0.0980 ^$^
BMI (kg/m^2^)	26.4 (14.2–54.3)	26.2 (15.7–54.3)	28.1 (14.2–45.4)	0.5876 ^$^
BMI SDS	2.1 (−2.0–5.7)	1.8 (−1.3–5.0)	2.5 (−2.0–5.7)	0.1351 ^$^
Obese n (%)	70 (56.4%)	29 (47.5%)	41 (65.1%)	0.07377 ^&^
Glycemia (mg/dL)	80.0 (60.0–167.0)	78.0 (60.0–167.0)	83.0 (63.0–163.0)	0.0128 ^$^ *
Insulin (µU/mL)	11.4 (0.5–49.4)	9.8 (0.5–49.4)	12.2 (2.7–46.5)	0.1990 ^$^
HOMA	2.3 (0.1–10.2)	1.9 (0.1–9.6)	2.7 (0.4–10.2)	0.1207 ^$^
HbA1c	5.4 (4.3–9.9)	5.3 (4.6–9.9)	5.4 (4.3–8.2)	0.3014 ^$^
Total cholesterol (mg/dL)	166.5 (106.0–256.0)	163.0 (106.0–243.0)	171.0 (113.0–256.0)	0.7003 ^$^
HDL-C (mg/dL)	52.0 (32.0–107.0)	54.0 (32.0–107.0)	49.0 (36.0–96.0)	0.0687 ^$^
Triglycerides (mg/dL)	77.0 (30.0–266.0)	74.0 (35.0–218.0)	84.0 (30.0–266.0)	0.2114 ^$^
SBP (mm/Hg)	111.0 (80.0–141.0)	110.0 (80.0–138.0)	116.0 (89.0–141.0)	0.1081 ^$^
DBP (mm/Hg)	70.0 (40.0–92.0)	69.0 (40.0–92.0)	70.0 (50.0–89.0)	0.3021 ^$^
FFM (kg)	31.4 (11.4–65.8)	28.2 (11.4–50.8)	35.4 (14.2–65.8)	0.0039 ^$^ *
FFM (%)	53.6 (35.4–87.3)	51.5 (35.4–69.8)	54.9 (40.6–87.3)	0.0458 ^$^ *
FM (kg)	27.1 (2.3–71.2)	24.4 (7.0–71.2)	28.3 (2.3–62.1)	0.3763 ^$^
FM (%)	46.5 (12.7–64.6)	48.5 (30.2–64.6)	45.1 (12.7–59.4)	0.0458 ^$^ *
TMI	88.0 (20.9–200.8)	80.6 (20.9–200.8)	96.7 (25.3–196.6)	0.0437 ^$^ *
BMFI	10.7 (1.7–34.7)	10.4 (2.7–34.7)	10.9 (1.7–28.4)	0.8592 ^$^
FMI	11.9 (1.9–40.5)	12.1 (4.2–40.5)	11.6 (1.9–23.4)	0.9502 ^$^
FFMI	31.4 (11.4–65.8)	28.2 (11.4–50.8)	35.4 (14.2–65.8)	0.0039 ^$^ *
ABSI	0.1 (0.1–0.1)	0.1 (0.1–0.1)	0.1 (0.1–0.1)	0.0443 ^$^ *
VAI	1.0 (0.3–4.2)	1.1 (0.4–4.2)	0.9 (0.3–4.1)	0.1006 ^$^
WtHR	0.6 (0.4–0.9)	0.6 (0.4–0.9)	0.6 (0.4–0.9)	0.4732 ^$^
CMI	0.9 (0.3–5.7)	0.8 (0.3–3.1)	1.0 (0.3–5.7)	0.1006 ^$^
TC/HDL-C	3.2 (1.5–7.1)	3.0 (1.5–5.6)	3.4 (1.7–7.1)	0.0236 ^$^ *
TG/HDL-C	1.5 (0.5–7.0)	1.5 (0.5–5.2)	1.7 (0.5–7.0)	0.0748 ^$^
MetS %	19.4	14.8	23.8	0.2020 ^&^

Abbreviations: WC: waist circumference; BMI: Body Mass Index; BMI SDS: BMI standard deviation score; HbA1c: glycated hemoglobin; HDL-C: high-density lipoprotein; SBP: systolic blood pressure; DBP: diastolic blood pressure; FFM: fat free mass; FM: fat mass; TMI: tri-ponderal mass index; BMFI: body mass fat index; FMI: fat mass index; FFMI: fat-free mass index; ABSI: body shape index; VAI: visceral adiposity index; WtHR: waist-to-height ratio; CMI: cardiometabolic index; TC/HDL-C: total cholesterol/HDL-C ratio; TG/HDL-C: triglycerides/HDL-C ratio: MetS: metabolic syndrome. For the difference between females vs. males, *: *p* < 0.05; ^$^: Wilcoxon test; ^&^: chi-square test.

**Table 3:** Clinical and biochemical data subdivided according to the presence/absence of MetS. Data are expressed as median (interquartile range).

Variable	Total (n = 124)	MetS (n = 24)	No MetS (n = 100)	*p*
Age (yr)	13.3 (6.9–18.9)	12.0 (7.0–18.2)	14.0 (6.9–18.9)	0.1799 ^$^
Weight (kg)	60.1 (19.0–121.7)	69.4 (27.8–121.7)	58.5 (19.0–116.0)	0.0417 ^$^ *
Height (cm)	149.3 (108.0–174.7)	144.1 (122.4–166.5)	150.4 (108.0–174.7)	0.4846 ^$^
WC (cm)	86.5 (54.0–131.0)	104.5 (61.5–131.0)	83.5 (54.0–117.0)	0.0010 ^$^ *
BMI (kg/m^2^)	26.4 (14.2–54.3)	36.2 (17.3–54.3)	25.6 (14.2–49.7)	0.0021 ^$^ *
BMI SDS	2.1 (−2.0–5.7)	3.5 (0.9–5.7)	1.9 (−2.0–4.7)	<0.0001 ^$^ *
Obese n (%)	70 (56.4%)	19 (79.2%)	51 (51.0%)	0.02321 ^&^
Glycemia (mg/dL)	80.0 (60.0–167.0)	86.0 (72.0–167.0)	79.5 (60.0–99.0)	0.0014 ^$^ *
Insulin (µU/mL)	11.4 (0.5–49.4)	17.8 (2.7–46.5)	9.8 (0.5–49.4)	0.0005 ^$^ *
HOMA	2.3 (0.1–10.2)	3.6 (0.6–10.2)	1.9 (0.1–9.6)	0.0001 ^$^ *
HbA1c	5.4 (4.3–9.9)	5.5 (4.7–9.9)	5.3 (4.3–9.4)	0.0566 ^$^
Total cholesterol (mg/dL)	166.5 (106.0–256.0)	172.5 (115.0–235.0)	165.5 (106.0–256.0)	0.9975 ^$^
HDL-C (mg/dL)	52.0 (32.0–107.0)	39.0 (32.0–72.0)	54.0 (36.0–107.0)	<0.0001 ^$^ *
Triglycerides (mg/dL)	77.0 (30.0–266.0)	133.0 (67.0- 266.0)	72.0 (30.0–202.0)	<0.0001 ^$^ *
SBP (mm/Hg)	111.0 (80.0–141.0)	114.5 (96.0–141.0)	111.0 (80.0–140.0)	0.3185 ^$^
DBP (mm/Hg)	70.0 (40.0–92.0)	75.0 (52.0–92.0)	69.0 (40.0–91.0)	0.0156 ^$^ *
FFM (kg)	31.4 (11.4–65.8)	33.6 (17.1–56.2)	30.9 (11.4–65.8)	0.0962 ^$^
FFM (%)	53.6 (35.4–87.3)	49.9 (35.4–69.5)	54.2 (40.6–87.3)	0.0369 ^$^ *
FM (kg)	27.1 (2.3–71.2)	33.7 (8.1–71.2)	24.7 (2.3–56.7)	0.0152 ^$^ *
FM (%)	46.5 (12.7–64.6)	50.2 (30.5–64.6)	45.8 (12.7–59.4)	0.0369 ^$^ *
TMI	88.0 (20.9–200.8)	96.4 (34.0–200.8)	86.0 (20.9–189.1)	0.1001 ^$^
BMFI	10.7 (1.7–34.7)	18.8 (3.5–34.7)	9.5 (1.7–29.8)	0.0010 ^$^ *
FMI	11.9 (1.9–40.5)	16.6 (5.4–40.5)	11.0 (1.9–25.3)	0.0027 ^$^ *
FFMI	31.4 (11.4–65.8)	33.6 (17.1–56.2)	30.9 (11.4–65.8)	0.0962 ^$^
ABSI	0.1 (0.1–0.1)	0.1 (0.1–0.1)	0.1 (0.1–0.1)	0.4003 ^$^
VAI	1.0 (0.3–4.2)	2.1 (0.6–4.2)	0.9 (0.3–2.4)	<0.0001 ^$^ *
WtHR	0.6 (0.4–0.9)	0.7 (0.5–0.9)	0.6 (0.4–0.8)	0.0001 ^$^ *
CMI	0.9 (0.3–5.7)	2.5 (0.5–5.7)	0.8 (0.3–2.4)	<0.0001 ^$^ *
TC/HDL-C	3.2 (1.5–7.1)	4.0 (2.6–5.6)	3.0 (1.5–7.1)	<0.0001 ^$^ *
TG/HDL-C	1.5 (0.5–7.0)	3.4 (1.1–7.0)	1.4 (0.5–4.0)	<0.0001 ^$^ *

Abbreviations: WC: waist circumference; BMI: Body Mass Index; BMI SDS: BMI standard deviation score; HbA1c: glycated hemoglobin; HDL-C: high-density lipoprotein; SBP: systolic blood pressure; DBP: diastolic blood pressure; FFM: fat free mass; FM: fat mass; TMI: tri-ponderal mass index; BMFI: body mass fat index; FMI: fat mass index; FFMI: fat-free mass index; ABSI: body shape index; VAI: visceral adiposity index; WtHR: waist-to-height ratio; CMI: cardiometabolic index; TC/HDL-C: total cholesterol/HDL-C ratio; TG/HDL-C: triglycerides/HDL-C ratio: MetS: metabolic syndrome. For the difference in Mets vs. No MetS, *: *p* < 0.05; ^$^: Wilcoxon test; ^&^: chi-square test.

**Table 4:** Clinical and biochemical data subdivided according to the presence of MetS (MetS +) and absence (MetS −) in the two genders. Data are expressed as median (interquartile range).

	MetS + (n = 24)			MetS − (n = 100)		
Variable	Females (n = 9)	Males (n = 15)	*p*	Females (n = 52)	Males (n = 48)	*p*
Age (yr)	11.1 (8.0–17.6)	13.2 (7.0–18.2)	0.8346 ^$^	13.2 (7.0–18.9)	14.5 (6.9–18.8)	0.6072 ^$^
Weight (kg)	66.2 (27.8–121.7)	79.2 (31.2–120.6)	0.4561 ^$^	57.4 (19.0–100.2)	60.0 (23.0–116.0)	0.2450 ^$^
Height (cm)	143.1 (122.4–165.0)	148.1 (124.0–166.5)	0.5709 ^$^	145.0 (108.0–173.1)	154.0 (110.1–174.7)	0.0033 ^$^ *
WC (cm)	91.0 (62.0–125.0)	110.5 (61.5–131.0)	0.2570 ^$^	82.0 (54.0–115.0)	86.8 (56.0–117.0)	0.41951 ^$^
BMI (kg/m^2^)	28.1 (18.6–54.3)	38.5 (17.3–45.4)	0.5711 ^$^	25.8 (15.7–49.7)	25.1 (14.2–43.7)	0.7146 ^$^
BMI SDS	2.7 (1.1–5.0)	3.6 (0.9–5.7)	0.1997 ^$^	1.8 (−1.3–4.7)	2.1 (−2.0–3.8)	0.6097 ^$^
Obese n (%)	5 (55.5%)	14 (93.3%)	0.09158 ^&^	24 (46.1%)	27 (56.2%)	0.41863 ^&^
Glycemia (mg/dL)	80.0 (72.0–167.0)	88.0 (77.0–163.0)	0.1353 ^$^	78.0 (60.0–99.0)	81.5 (63.0–99.0)	0.0940 ^$^
Insulin (µU/mL)	15.5 (7.3–35.9)	17.9 (2.7–46.5)	0.8815 ^$^	8.7 (0.5–49.4)	10.4 (2.7–37.0)	0.2593 ^$^
HOMA	3.6 (1.4–6.4)	3.6 (0.6–10.2)	0.9287 ^$^	1.6 (0.1–9.6)	2.0 (0.4–7.4)	0.1698 ^$^
HbA1c	5.3 (5.0–9.9)	5.5 (4.7–8.2)	0.5107 ^$^	5.3 (4.6–9.4)	5.4 (4.3–6.1)	0.6937 ^$^
Total cholesterol (mg/dL)	156.0 (125.0–235.0)	179.0 (115.0–216.0)	0.2700 ^$^	164.0 (106.0–243.0)	168.0 (113.0–256.0)	0.9917 ^$^
HDL-C (mg/dL)	38.0 (32.0–72.0)	41.0 (36.0–54.0)	0.0586 ^$^	56.5 (38.0–107.0)	54.0 (36.0–96.0)	0.0710 ^$^
Triglycerides (mg/dL)	130.0 (71.0–218.0)	146.0 (67.0–266.0)	0.4929 ^$^	71.0 (35.0–202.0)	75.5 (30.0–161.0)	0.4901 ^$^
SBP (mm/Hg)	110.0 (98.0–127.0)	120.0 (96.0–141.0)	0.2826 ^$^	110.0 (80.0–138.0)	115.0 (89.0–140.0)	0.2373 ^$^
DBP (mm/Hg)	75.0 (52.0–92.0)	76.0 (54.0–85.0)	0.3864 ^$^	68.5 (40.0–91.0)	70.0 (50.0–89.0)	0.6502 ^$^
FFM (kg)	29.9 (18.5–50.8)	42.2 (17.1–56.2)	0.2966 ^$^	27.9 (11.4–46.1)	34.7 (14.2–65.8)	0.0107 ^$^ *
FFM (%)	50.2 (35.4–69.5)	49.8 (43.6–64.4)	0.9762 ^$^	53.1 (40.9–69.8)	57.6 (40.6–87.3)	0.0100 ^$^ *
FM (kg)	31.3 (8.1–71.2)	34.7 (11.2–62.1)	0.5312 ^$^	24.3 (7.0–51.1)	25.8 (2.3–56.7)	0.7667 ^$^
FM (%)	49.8 (30.5–64.6)	50.2 (35.6–56.4)	0.9762 ^$^	46.9 (30.2–59.1)	42.4 (12.7–59.4)	0.0100 ^$^ *
TMI	95.3 (34.0–200.8)	125.0 (41.9–196.6)	0.4561 ^$^	80.4 (20.9–147.7)	95.4 (25.3–189.1)	0.0923 ^$^
BMFI	12.7 (3.5–34.7)	21.5 (3.8–28.4)	0.6547 ^$^	10.3 (2.7–29.8)	8.7 (1.7–25.0)	0.3961 ^$^
FMI	13.1 (5.4–40.5)	19.1 (6.2–23.4)	0.7429 ^$^	11.9 (4.2–25.3)	10.6 (1.9–21.4)	0.3238 ^$^
FFMI	29.9 (18.5–50.8)	42.2 (17.1–56.2)	0.2966 ^$^	27.9 (11.4–46.1)	34.7 (14.2–65.8)	0.0107 ^$^ *
ABSI	0.1 (0.1–0.1)	0.1 (0.1–0.1)	0.2700 ^$^	0.1 (0.1–0.1)	0.1 (0.1–0.1)	0.1239 ^$^
VAI	2.6 (0.8–4.2)	2.0 (0.6–4.1)	0.1011 ^$^	1.0 (0.4–2.4)	0.8 (0.3–2.2)	0.0109 ^$^ *
WtHR	0.7 (0.5–0.9)	0.7 (0.5–0.9)	0.3252 ^$^	0.6 (0.4–0.8)	0.6 (0.4–0.8)	0.7510 ^$^
CMI	2.5 (0.5–3.1)	2.4 (0.6–5.7)	0.6983 ^$^	0.7 (0.3–1.8)	0.8 (0.3–2.4)	0.3410 ^$^
TC/HDL-C	3.9 (3.3–5.6)	4.0 (2.6–5.2)	0.6983 ^$^	2.9 (1.5–4.6)	3.2 (1.7–7.1)	0.0401 ^$^ *
TG/HDL-C	3.4 (1.1–5.2)	3.3 (1.3–7.0)	0.9762 ^$^	1.4 (0.5–3.0)	1.4 (0.5–4.0)	0.1807 ^$^

Abbreviations: WC: waist circumference; BMI: Body Mass Index; BMI SDS: BMI standard deviation score; HbA1c: glycated hemoglobin; HDL-C: high-density lipoprotein; SBP: systolic blood pressure; DBP: diastolic blood pressure; FFM: fat-free mass; FM: fat mass; TMI: tri-ponderal mass index; BMFI: body mass fat index; FMI: fat mass index; FFMI: fat-free mass index; ABSI: body shape index; VAI: visceral adiposity index; WtHR: waist-to-height ratio; CMI: cardiometabolic index; TC/HDL-C: total cholesterol/HDL-C ratio; TG/HDL-C: triglycerides/HDL-C ratio: MetS: metabolic syndrome. For the difference between females vs. males, *: *p* < 0.05; ^$^: Wilcoxon test; ^&^: chi-square test.

**Table 5:** Sensitivity, specificity, positive predictive value, negative predictive value, Youden Index, and Area Under the Receiver Operating Characteristic curve of CMI and TG/HDL-C ratio in the whole study group and the two genders. Data are expressed as median (interquartile range) when appropriate.

CMI	Whole Group	Females	Males
Sensitivity	0.7083 (0.5265–0.8902)	0.7778 (0.5062–1.000)	0.6667 (0.4281–0.9052)
Specificity	0.9800 (0.9526–1.000)	1.000 (1.000–1.000)	0.9583 (0.9018–1.000)
PPV	0.8947 (0.7567–1.000)	1.000 (1.000–1.000)	0.8333 (0.6225–1.000)
NPV	0.9333 (0.8856–0.9810)	0.9630 (0.9126–1.000)	0.9020 (0.8203–0.9836)
Youden Index	0.773	0.778	0.783
AUROC	0.906	0.887	0.910 *
TG/HDL-C	Whole group	Females	Males
Sensitivity	0.6250 (0.4313–0.8187)	0.6667 (0.3587–0.9746)	0.6000 (0.3521–0.8479)
Specificity	0.9700 (0.9366–1.000)	0.9808 (0.9434–1.000)	0.9583 (0.9018–1.000)
PPV	0.8333 (0.6612–1.000)	0.8571 (0.5979–1.000)	0.8182 (0.5903–1.000)
NPV	0.9151 (0.8620–0.9682)	0.9444 (0.8833–1.000)	0.8846 (0.7978–0.9715)
Youden Index	0.723	0.759	0.721
AUROC	0.905	0.912	0.894 *

Abbreviations: CMI: cardiometabolic index; TG/HDL-C: triglycerides/HDL-C ratio; PPV: positive predictive value; NPV: negative predictive value; AUROC: Area Under the Receiver Operating Characteristic curve. For significance: * *p* < 0.05 compared to the other index.

**Table 6:** Bivariate correlation coefficients between TG/HDL-C ratio and the components of MetS.

	Whole Group	Females	Males
BMI (kg/m^2^)	0.36 *	0.30 *	0.42 *
BMI SDS	0.36 *	0.33 *	0.42 *
glycemia (mg/dL)	0.22 *	0.24	0.17
HDL-C (mg/dL)	−0.69 *	−0.65 *	−0.73 *
Triglycerides (mg/dL)	0.90 *	0.85 *	0.93 *
SBP (mm/Hg)	0.22 *	0.09	0.30 *
DBP (mm/Hg)	0.21 *	0.16	0.23

Abbreviations: BMI: Body Mass Index; SDS: standard deviation score; HDL: high-density lipoprotein; SBP: systolic blood pressure; DBP: diastolic blood pressure; TG/HDL-C ratio: triglyceride-to-high-density lipoprotein ratio. For significance: * *p* < 0.05.

## Data Availability

The datasets used and/or analyzed in the present study will be uploaded to www.zenodo.org (accessed on 27 June 2025) and made available by the corresponding author upon a reasonable request.

## Abbreviations

The following abbreviations are used in this manuscript:

ABSI	body shape index
AUC	area under the curve
AUROC	Area Under the Receiver Operating Characteristic Curve
BMI	Body Mass Index
BMFI	body mass fat index
BP	blood pressure
CI	confidence interval
CMI	cardiometabolic index
CVD	cardiovascular disease
del15	paternal deletion of chromosome 15
DXA	dual-energy X-ray absorptiometry
EC	Ethical Committee
FFM	fat-free mass
FFMI	fat-free mass index
FM	fat mass
FMI	fat mass index
FPG	fasting plasma glucose
HbA1c	hemoglobin A1c
HDL-C	high-density lipoprotein cholesterol
HOMA	homeostatic model assessment
IDEFICS	Identification and prevention of Dietary- and lifestyle-induced health EFfects In Children and infantS
IDF	International Diabetes Federation
IQR	interquartile range
IR	insulin resistance
MetS	metabolic syndrome
NLR	negative likelihood ratio
NPV	negative predictive value
PLR	positive likelihood ratio
PPV	positive predictive value
PWS	Prader–Willi syndrome
rhGH	recombinant human growth hormone
ROC	receiver operating characteristic
TC	total cholesterol
T2DM	type 2 diabetes mellitus
TG	triglyceride
TMI	tri-ponderal mass index
UPD15	maternal uniparental disomy for chromosome 15
VAI	visceral adiposity index
WC	waist circumference
WHO	World Health Organization
WtHR	waist-to-height ratio
